# A digital assessment system for evaluating kinetic tremor in essential tremor and Parkinson’s disease

**DOI:** 10.1186/s12883-018-1027-2

**Published:** 2018-03-09

**Authors:** Po-Chieh Lin, Kai-Hsiang Chen, Bing-Shiang Yang, Yu-Jung Chen

**Affiliations:** 1 Department of Mechanical Engineering, National Chiao Tung University, 1001 University Road, Hsinchu City, 30010 Taiwan; 2 Neurology Division, National Taiwan University Hospital, Hsinchu Branch, Hsinchu City, 300 Taiwan; 3 Institute of Biomedical Engineering, National Chiao Tung University, Hsinchu City, 300 Taiwan

**Keywords:** Essential tremor, Parkinson’s disease, Spiral drawing, Clinical assessment

## Abstract

**Background:**

Spiral drawing on papers is a common tremor evaluation tool for diagnosing patients with essential tremor (ET) or Parkinson’s disease (PD). No standard drawing methods and parameters that use graphic tablets are yet available for objective evaluation.

**Methods:**

This study established a tremor assessment system for tremor severity by using graphic tablets. Twelve patients with ET and twelve patients with PD were tested to establish system algorithms, and six additional patients were tested with the developed system to evaluate its performance. The patients also performed spiral drawing with three guiding paradigms on a graphic tablet: traced along a given spiral (S1), performed freehand drawing (S2), and traced along a guiding point (S3). Three parameters were calculated to quantify tremor severity: the means of radial difference per radian (*|*dr/dθ*|*), the means of radial difference per second (*|*dr/dt*|*), and the area under curve (AUC) of the frequency spectrum of the velocity. Each patient’s drawing was also evaluated using a visual rating scale (VRS) by experienced physicians. The interrater reliability was examined to identify the most consistent test paradigm.

**Results:**

The parameter *|*dr/dθ*|* and AUC correlated well with the VRS (*R* > 0.8) in S1, S2 and S3 tests. The S1 test presented the best interrater reliability (Weighted Kappa coefficient, k = 0.80) among three tests. The Weighted Kappa coefficients are 0.67 and 0.71 in S2 and S3 tests, respectively.

**Conclusions:**

We developed three different guiding paradigms for spiral drawing on a digital graphic tablet for clinical tests. Three parameters were calculated to represent the tremor severity in spiral drawing and used to quantify temporal and spatial characteristics of tremor, and provided good correlation with current clinical assessments. The test “traced along a given spiral” is recommended due to its good interrater reliability.

**Electronic supplementary material:**

The online version of this article (10.1186/s12883-018-1027-2) contains supplementary material, which is available to authorized users.

## Background

Essential tremor (ET) and Parkinson’s disease (PD) are the two most common tremor disorders, and their prevalence increases with age. Although a tremor does not pose an immediate threat to life, it affects the quality of life. In more than 75% of patients, daily-life activities such as writing and eating are affected [1]. In clinical practice, a kinetic tremor is mainly assessed with writing or spiral drawing, and a score of 0 to 9 on a visual rating scale (VRS) is often used to evaluate the spiral drawing and determine the severity of the tremor [2]. This score is also used as a clinical index for comparing studies based on the quantification of a tremor [3–5]. Although clinicians receive training, studies have shown that owing to the difference between the adjacent scores, the clinical scales may not be able to distinguish subtle differences in tremor severity or show changes in tremor severity within a certain time period [6]. Thus, an objective and detailed description of the severity or efficacy of drug treatment cannot be readily achieved. Therefore, more objective tools, such as using a graphics tablet for quantifying the severity of a tremor, are required for clinical research even in long-term care. Several studies have used graphic tablets for disease severity assessments. A previous study asked ET patients to draw spirals on a graphic tablet, and the graphs were recorded and then analyzed offline [7]. A multiorder differential parameter of the radius per radian was calculated and correlated well with the tremor severity quantified by clinical assessments [8]. Pullman used indices related to spirogram spatial irregularity, including first- and second-order smoothness, zero crossing, and tightness, for comparison with clinical rating scales using artificial neural networks. The results showed good correlation to motor symptom severity of PD [9], a disease that is often misdiagnosed with ET [10, 11]. Louis et al. used the variability in the width of each circle in the spiral as a tremor severity index for determining ET severity [4].

In recent years, because graphics tablets are incorporated with LCD screens, patients can draw on the graphic tablets directly with visuomotor feedback. A spectrum analysis for these Archimedes spirals on the graphic tablets was used to determine the major tremor frequency, and the area under the curve (AUC) of ±1 Hz range next to the peak frequency was also used as an index to represent tremor severity. This index showed positive logarithmic correlation to VRS [3]. Although the spiral drawing method has been widely used in clinical practice, unlike spirogram drawing on papers, there is still a lack of suggestive standards for assessing the spiral drawings on graphic tablets [5, 12, 13]. Moreover, a previous study reported that the interrater reliability of VRS assessment determined using freehand drawing is different from that determined using tracing a simple spiral [14]. In addition, the drawing velocity is not standardized. Furthermore, some recent studies have attempted to quantify the tremor characteristics to decrease the misdiagnosis rate [4, 15]. Because tremor-related diseases result in progressive neurological degeneracy, a real-time assessment system should be implemented for evaluating the severity and characteristics of tremors for home or institutional care, especially in the elderly.

Therefore, we aimed to develop a tremor assessment system with testing paradigms on a graphic tablet with suitable parameters for automatically quantifying tremor characteristics and severity in real time. In this study, we used three Archimedes spiral templates (tracing a sample spiral, freehand drawing, and tracing a guiding point with a constant velocity) on a graphic tablet and three assessing parameters to correlate with clinical rating scales, that is, VRS scales. The interrater reliability between clinical assessments by experienced physicians (neurologists) was also evaluated. In addition, the system was validated using six additional subjects (validation group).

## Methods

**Fig. 1:**
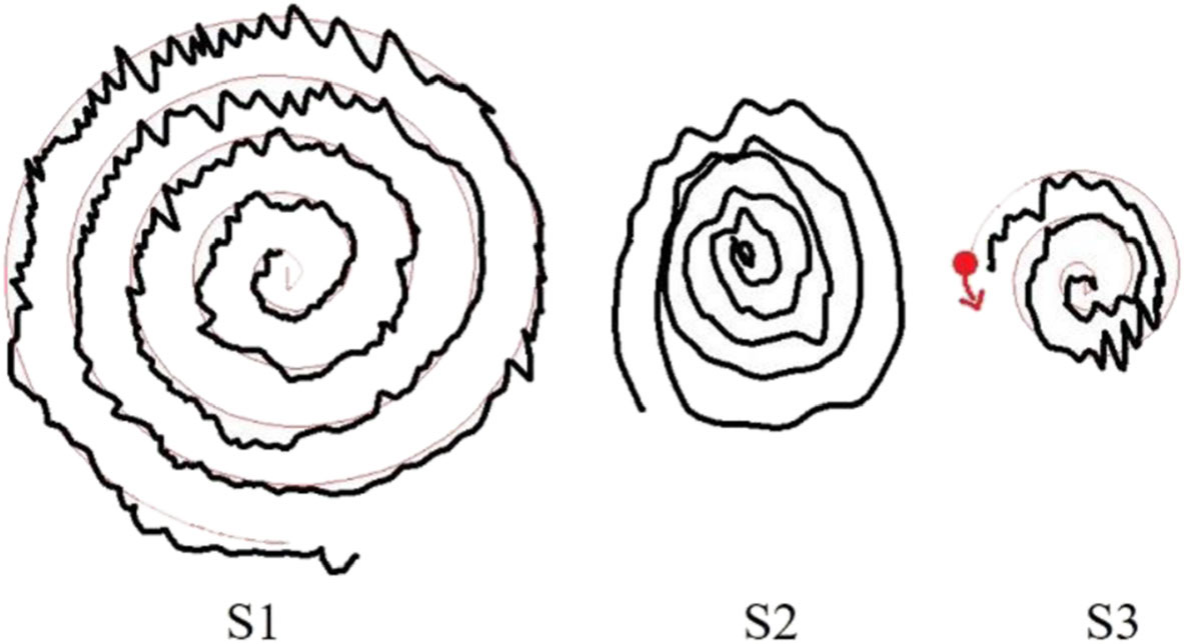
Three testing conditions on the graphic tablet. In S1, the subject was instructed to follow the thin line and start from the center of the spiral. The thin line was the target spiral and the thick line was the trace by the patient. In S2, the patients were instructed to draw the spiral on a blank graphic tablet without any constraints (with freehand). The thick line was the drawing. In S3, the subject was instructed to follow a thin guiding point. The guiding point moved at a constant speed. The thick line was the trace by the subject and the thin line was the trajectory of the guiding point

To build the tremor assessment system, we used a Wacom (Cintiq 13HD) graphic tablet (size: 14.75 × 9.75 × 0.5 in; screen: 13.3 in; resolution: 5080 dpi; capture frequency: 50 Hz) for different spiral drawing tests and recording the drawing trajectory. Three spiral drawing conditions (with different guiding paradigms) were generated by a custom-made computer program: S1, S2, and S3 (Fig. 1).

In S1, the patients traced along a given spiral. In S2, the patients were asked to draw spirographs freely. In S3, the patients were asked to follow a guiding point moving at a fixed speed to complete a spirograph (the completed spiral is the same as that in S1). Patients were instructed to practice the drawing in three tests until they were familiarized with the platform and conditions. The maximum radius of the spiral was 30 mm, with an interloop distance of 6 mm. At the start of the S1 test, a standard spiral graph was created on the touch screen. Each subject (described herein) was asked to draw from the center of an indicated circle using a stylus touch pen and try to follow the standard spiral graph (a thin line) to complete all the paths. In the S2 test, the subject was asked to draw small to large spiral circles on the touch screen without time or space specifications. In the S3 test, a guiding point was generated on the tablet, and the subject was asked to follow the guiding point to complete the spirograph.

In this study, a total of 30 subjects were recruited: 12 patients (aged 71.8 ± 6.2 years) with ET, 12 patients (aged 67.3 ± 5.3 years) with PD (testing group; total of 24 patients); and six additional patients (aged 67.6 ± 6.5 years) with ET or PD (validation group). The aforementioned three tests (S1, S2, and S3) were performed by every subject three times each with the left and right hands, respectively. The VRS of each drawing by the subjects was graded blindly by three experienced neurologists. The patients were instructed to take half of their regular dose of the prescribed antitremor medication one time before the test. This allowed the patients to present with symptoms without considerably affecting their daily activities. The experimental procedures were approved by the Institutional Review Board of the National Taiwan University Hospital, Hsinchu Branch. Every subject signed an informed consent form before the test.

**Fig. 2:**
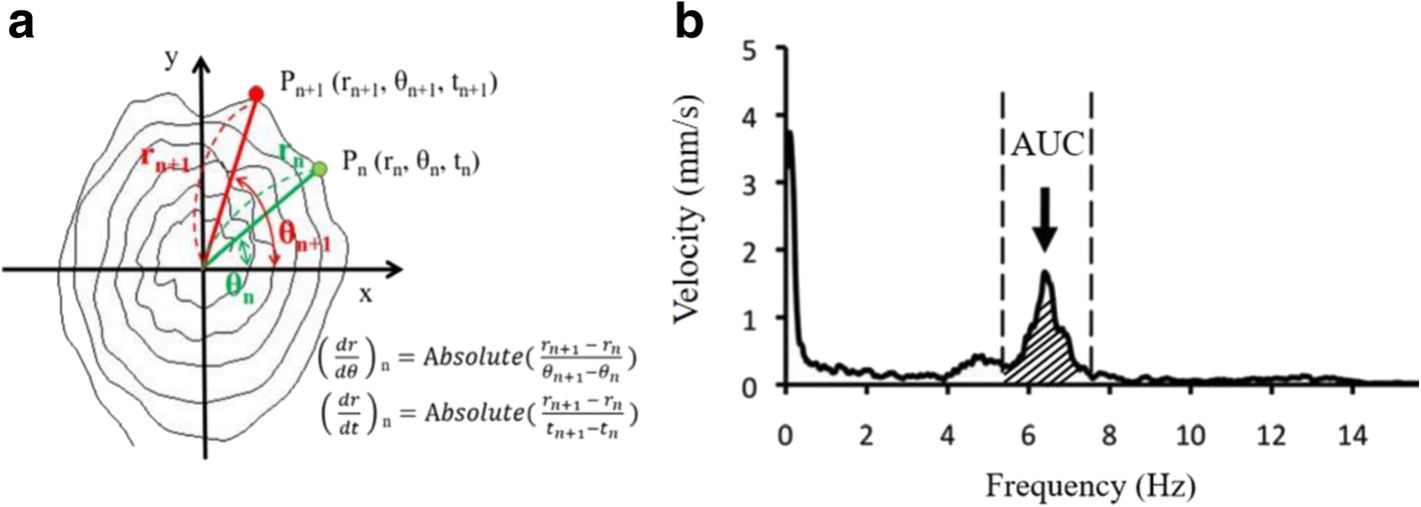
**a** Position of the spiral graphs defined in polar coordinates, and the definitions of the *|*dr/dθ*|* and *|*dr/dt*|*. **b** The figure shows the velocity spectrum. The AUC was define as the area of the range between ±1 Hz of main frequency of velocity spectrum

A rapid increase or decrease in the radius of the spiral may appear in the spiral drawings by the patients. The variations or changes in the radius represent the tremor severity. A polar coordinate system was used to describe the trace of the spiral drawing (Fig. 2). The spiral trajectory position P (r, θ, t) was expressed in a two-dimensional plane, and the starting point of the spiral graph was set as the coordinate origin O (0, 0, 0), where r is the radius and θ is the angle (in radius or rad). In this study, the tremor characteristics and severity were quantified by three parameters (*|*dr/dt*|*, *|*dr/dθ*|*, and AUC) calculated from the spiral drawings, and their correlations with the VRS scores were also determined.

To quantify the drawing characteristics, several parameters of the subject’s drawing were calculated. |dr/dt| was defined as the absolute radial distance divided by the time period between two consecutive samples. The average of all the |dr/dt| values throughout out a drawing trial was then computed and the average of the three computed values (from three trials in each condition respectively) was defined as ‘mean of |dr/dt|’. *|*dr/dθ*|* was defined as the absolute value of radial distance divided by the angular difference (in rad) between two consecutive samples. The average of all the *|*dr/dθ*|* throughout out a drawing trial was then calculated and then the average of the three calculated values (from three trials in each condition respectively) was defined as ‘mean of *|*dr/dθ*|*’ (see Fig. 2a). The third parameter, AUC, was adopted from a previous study [3] by calculating the AUC of ±1 Hz range next to the peak frequency of the power spectrum of the drawing velocity (Fig. 2b). The average AUC value of three trials in each condition will be presented as a single AUC in later text. After the patient completed the spiral graphs, each graph was graded by three neurologists independently and blindly using the VRS. The mean value of the three VRS scores was then calculated to determine the clinical tremor severity in each condition. Because a previous study reported that only the logarithmic value of the amplitude was linearly correlated with the clinical evaluation scores [16], in this study, the Pearson correlation coefficient was used to measure the strength of a linear association between the logarithmic values of the three parameters and the clinical tremor severity (mean VRS scores) by using SPSS22 among the three spiral drawing tests (S1, S2, and S3). Moreover, to examine the effects of the interrater reliability between the physicians, the Weight Kappa of the testing group was calculated. To verify the system efficacy, the VRS difference between the clinical raters and our system was calculated. The averaged VRS (VRSavg) evaluated by the three raters was also calculated as a reference. The VRSd-rater was defined as the average of absolute difference between each rater and the VRSavg.

## Results

In this study, we developed a tremor assessment system, and the correlations coefficients, the interrater reliability between the raters, and the validation of the system are described as follows.

### Correlation coeffecints

**Fig. 3:**
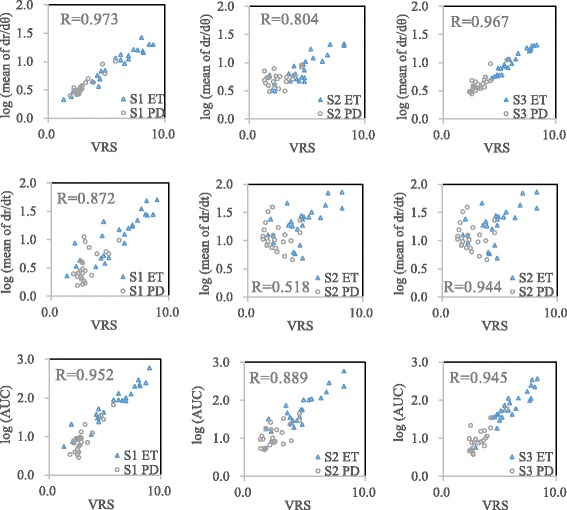
Linear regression analysis of the logarithm values of the three investigated parameters and VRS scores in the three test conditions. Data from 24 patients (testing group) were included in each figure, and each patient had two data points (two hands). The parameters log(*|*dr/dθ*|*) and log(AUC) correlated well with the VRS scores (*R* > 0.7) regardless of the test conditions, and all correlations were statistically significant (*p* < 0.01) (the units of *|*dr/dt*|*: mm/s; *|*dr/dθ*|*: mm/rad; AUC: mm × Hz/s)

The relationships between VRS and the three investigated parameters of the data from the testing group (24 patients) are shown in Fig. 3. The linear correlation coefficients with VRS in the S1 test were 0.973 for log (*|*dr/dθ*|*), 0.872 for log (*|*dr/dt*|*), and 0.952 for log (AUC) (all *p* < 0.01).

The correlation coefficients in the S2 test were 0.804 for log (*|*dr/dθ*|*), 0.518 for log (*|*dr/dt*|*), and 0.889 for log (AUC). In the S2 test, log (*|*dr/dθ*|*) and log (AUC) showed a high correlation (*p* < 0.01); log (*|*dr/dt*|*) showed a medium correlation with the VRS (*p* < 0.01).

In the S3 test, the correlation coefficients were 0.967 for log (*|*dr/dθ*|*), 0.944 for log (*|*dr/dt*|*), and 0.945 for log (AUC). All the parameters showed a high correlation (*R* > 0.7; *p* < 0.01). Overall, log (*|*dr/dθ*|*) and log (AUC) correlated well with the VRS scores irrespective of the test conditions.

### Interrater reliability

The Weighted Kappa method was used to evaluate the interrater reliability among VRS rated by the three raters of the three tests of the testing group. The S1 test had the highest interrater reliability among the three tests (see Table 1).

**Table 1:** Interrater reliability of different spiral drawing paradigms (Weighted Kappa)

Rater/Weighted Kappa	S1	S2	S3
Rater 1, 2	0.83	0.78	0.74
Rater 2, 3	0.79	0.56	0.60
Rater 1, 3	0.78	0.68	0.79
Average	0.80	0.67	0.71

### System validation

Because one spiral drawing was rated by three raters independently, the VRS values may be different among the three raters. Therefore, the absolute VRS difference (VRSd-rater) between the VRS evaluated by each rater and the mean VRS of three raters (VRSavg) was calculated. The mean VRSavg of the testing group were shown in Table 2, and the S1 test had the minimum interrater difference, 0.65, which means the VRS values evaluated by the raters were the closest among three tests.

**Table 2:** The absolute VRS difference (VRSd-rater) between the VRSs evaluated by each rater and the mean VRSs of the three raters (VRSavg) of testing group

	S1	S2	S3
VRSd-rater	0.65	0.83	0.73

The correlated VRS (cVRS) was calculated using the linear function obtained from the linear regression. The functions of each test are as follows:

$$ \mathrm{S}1:\mathrm{cVRS}\ \mathrm{from}\ \mathrm{mean}\ \mathrm{of}\mid \mathrm{dr}/\mathrm{d}\uptheta \mid =\left[\ \log \left(\mathrm{mean}\ \mathrm{of}\ |\mathrm{dr}/\mathrm{d}\uptheta |\right)\hbox{--} 0.1317\right]/0.1394 $$

$$ \mathrm{S}1:\mathrm{cVRS}\ \mathrm{from}\ \mathrm{mean}\ \mathrm{of}\mid \mathrm{dr}/\mathrm{dt}\mid =\left[\ \log \left(\mathrm{mean}\ \mathrm{of}\ |\mathrm{dr}/\mathrm{dt}|\right)\hbox{--} 0.0437\right]/0.1747 $$

$$ \mathrm{S}1:\mathrm{cVRS}\ \mathrm{from}\ \mathrm{AUC}=\left[\ \log \left(\mathrm{AUC}\right)\hbox{--} 0.1463\right]/0.2797 $$

$$ \mathrm{S}2:\mathrm{cVRS}\ \mathrm{from}\ \mathrm{mean}\ \mathrm{of}\mid \mathrm{dr}/\mathrm{d}\uptheta \mid =\left[\ \log \left(\mathrm{mean}\ \mathrm{of}\ |\mathrm{dr}/\mathrm{d}\uptheta |\right)\hbox{--} 0.4464\right]/0.0977 $$

$$ \mathrm{S}2:\mathrm{cVRS}\ \mathrm{from}\ \mathrm{mean}\ \mathrm{of}\mid \mathrm{dr}/\mathrm{dt}\mid =\left[\ \log \left(\mathrm{mean}\ \mathrm{of}\ |\mathrm{dr}/\mathrm{dt}|\right)\hbox{--} 0.8748\right]/0.0864 $$

$$ \mathrm{S}2:\mathrm{cVRS}\ \mathrm{from}\ \mathrm{AUC}=\left[\ \log \left(\mathrm{AUC}\right)\hbox{--} 0.5054\right]/0.2509 $$

$$ \mathrm{S}3:\mathrm{cVRS}\ \mathrm{from}\ \mathrm{man}\ \mathrm{of}\mid \mathrm{dr}/\mathrm{d}\uptheta \mid =\left[\ \log \left(\mathrm{mean}\ \mathrm{of}\ |\mathrm{dr}/\mathrm{d}\uptheta |\right)\hbox{--} 0.1536\right]/0.1418 $$

$$ \mathrm{S}3:\mathrm{cVRS}\ \mathrm{from}\ \mathrm{mean}\ \mathrm{of}\mid \mathrm{dr}/\mathrm{dt}\mid =\left[\log \left(\mathrm{mean}\ \mathrm{of}\ |\mathrm{dr}/\mathrm{dt}|\right)\hbox{--} 0.2892\right]/0.1521 $$

$$ \mathrm{S}3:\mathrm{cVRS}\ \mathrm{from}\ \mathrm{AUC}=\left[\log \left(\mathrm{AUC}\right)\hbox{--} 0.0707\right]/0.2904 $$

**Fig. 4:**
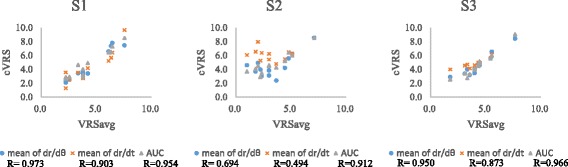
The correlation between VRSavg (evaluated by three physicians) and cVRS (based on from three parameters) of the validation group and its correlation coefficient. The result shows that cVRS correlate well with VRSavg in S1 and S3 test, especially based on *|*dr/dθ*|* in S1

**Fig. 5:**
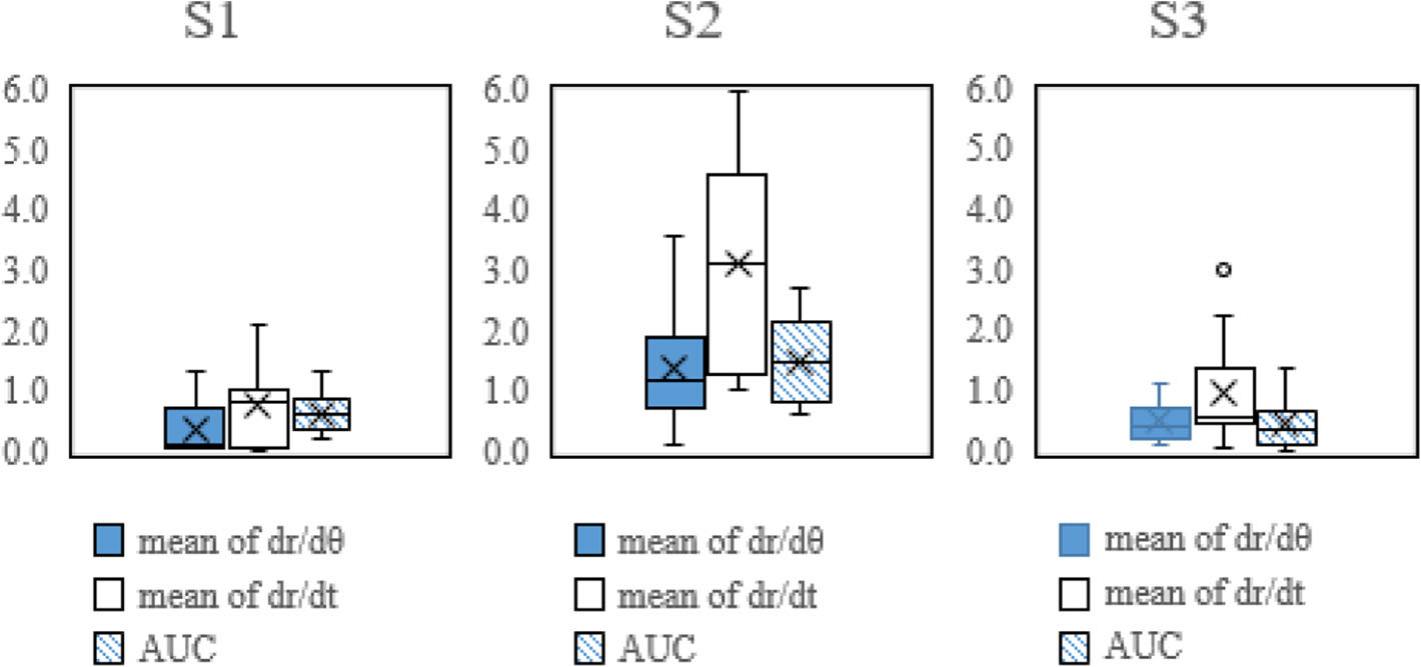
The box chart of the VRSd-system from three parameters of three tests. The variance of S1 and S3 test are much smaller than S2, indicating that S2 has the much larger individual difference of group

We calculated the cVRS value from each measured parameter for every test condition in the validation group (six patients). The correlation between the three investigated parameters and VRS obtained from the data of the validation group is shown in Fig. 4. The correlation coefficients were larger than 0.9 in S1 and S3 tests. Moreover, the *|*dr/dθ*|* shows the highest correlation coefficient (0.973) in S1 test. We used a parameter, VRSd-system, to show the bias of the cVRS compared with VRSavg (averaged VRS evaluated by three physicians). The VRSd-system was defined as the absolute difference between the cVRS and VRSavg (shown in Table 3). The variance of the VRSd-system calculated from each parameter of three tests is displayed as box plots in Fig. 5. The results also show that the variances of VRSd-system are low in S1 and S3.

**Table 3:** Absolute difference between correlated VRS (cVRS) and VRSavg (rated by three physicians) in the validation group

VRSd-system	S1	S2	S3
cVRS from *|*dr/dθ*|* vs. VRSavg	0.38	1.39	0.50
cVRS from *|*dr/dt*|* vs. VRSavg	0.75	3.09	0.96
cVRS from AUC vs. VRSavg	0.63	1.50	0.45
Average	0.59	1.99	0.64

## Discussion

Spiral drawing tests have not yet been standardized in current clinical practice. In this study, we established a tremor assessment system using a graphic tablet platform for spiral drawing tests with three paradigms, and used three temporal and spatial parameters to quantify the severity of the spiral drawing of patients with ET and PD. The data from 12 ET and 12 PD patients indicated high correlations between most of our developed parameters and VRS in the S1, S2 and S3 tests. In clinical practice, the consistency of an assessment is important. Among the three tests, the S1 test had the highest interrater reliability (the interrater reliability − Weighted Kappa in S1 was approximately 0.80, Table 1). A previous study, studying ET patients with dystonia, suggested that the S2 (freehand drawing) was suitable for clinical evaluation [14]. With different patients group, our study included both PD and ET patients.

In Table 2, the VRS difference between the three raters (VRSd-rater) was approximately 0.65 in S1, which was also the lowest among the three tests. To validate the system performance, we tested additional six patients with tremor (validation group) using our developed assessment system. The cVRS values calculated from the regression functions established by the testing group matched well with the VRS values assessed by the experienced physicians. As shown in Table 3, the VRS (a score of 0 to 9) difference between the cVRS evaluated by this system and the VRS evaluated by the physicians were approximately 0.59 in S1 and 0.64 in S3 test (based on the mean of *|*dr/dθ*|*), and this difference was the lowest among the three tests. According to the results of interrater reliability and system validation, we designed the S1 test to mimic the current clinical test with a standard spiral to follow. The developed assessment system with S1 paradigm can potentially replace the current clinical assessment drawing on papers.

In a clinical environment, patients are often asked to perform a freehand drawing on a paper and the physician then determines the tremor severity (VRS) based on the patient’s completed drawing with unaided eyes. Therefore, only spatial characteristics of the drawing can be observed. In our developed system, we investigated both spatial and temporal characteristics of spiral drawings. The *|*dr/dθ*|* represented the radius changes per radian and the *|*dr/dt*|* represented the radius changes per time period. Based on the regression functions generated by our study, a 1.3 mm/rad of *|*dr/dθ*|* or a 1.1 mm/s of *|*dr/dt*|* would be evaluated as 0 in VRS in S1 test. It indicates that our predicted VRS might have little bias when assessing spiral drawing from a person with little or without tremor. Similar phenomenon can also be seen in S2 and S3 tests. Besides, in current clinical practice, the drawing velocity of the spiral graphs drawn by patients is not standardized, that is, the radius increment per second of each circle is different between patients. The parameter *|*dr/dt*|* can reflect the influence of the radius per second; however, *|*dr/dθ*|* is independent of the drawing velocity. Because there was no time variable in the *|*dr/dθ*|* values, this parameter was expected to be close to observations in current clinical practice, in which the clinical ratings are always based on the completed drawings without any time variables. Therefore, the correlations between *|*dr/dθ*|* and the clinical VRS were better than correlations between *|*dr/dt*|* and the clinical VRS in three tests. Moreover, the study from Haubenberger et al. showed high correlation between AUC and VRS [3], and the correlation coefficients between *|*dr/dθ*|* and VRS in our study was as high as the those between AUC and VRS.

Our S1 (tracing along a given spiral) test included conditions of spiral drawings similar to the current clinical practice. S1 involved a stationary visual guidance to constrain the geometry of the spiral. The values of the investigated spatial parameters, *|*dr/dθ*|*, in S1 were highly correlated with the results of clinical assessments (VRS), indicating that the tests with specific parameters performed on a graphic tablet can be good alternatives to the current clinical assessments (drawing on papers) for determining tremor severity.

In addition to spatial parameters, taking advantage of digital graphic tablets, we quantified temporal characteristics of the tremor, such as *|*dr/dt*|* of the spiral drawing, which provide additional information related to the disease-affected or age-related movement control. With a stationary guidance in S1, *|*dr/dt*|* showed a high correlation with VRS; however, the correlations were low (*R* < 0.6) while drawing freely (in S2), which may be caused by drawing characteristics, for example, drawing an uneven-spaced spiral at various speeds, that cannot be easily observed while evaluation VRS ratings. Although the S3 test may not directly correspond to tremor severity in the current clinical assessment, it may be used to test other movement abilities, for example, accuracy of the eye–hand coordination affected by different diseases, injuries, or degeneration. Studies have shown that visual guidance may be used as a detection method for subsequent PD symptoms in patients with ET [17]. The visuomotor coordination during dynamically guided drawing may reveal the degeneration in different brain regions. It may also be able to provide physicians with a subsequent analysis of ET or for differential diagnosis.

The system was validated by calculating the difference between the tremor severity (cVRS) evaluated by our system using different characteristic parameters and VRS assessed by the physicians. The average difference (VRSd-system) was approximately 0.59 in S1, 1.99 in S2, and 0.64 in S3 among the validation group (Table 3). As a reference, the average difference in VRS values (VRSd-rater) of the testing group evaluated by the three raters was approximately 0.65–0.83 (Table 2). The VRS difference between our system and the physicians was not higher than VRS difference among the three experienced raters in S1 and S3, especially in S1, which also indicates that the S1 is the most consistent test with current clinical spiral test. Figure 5 also shows the low variation of individual difference in S1 and S3. Besides, there are high correlations between the VRS evaluated by raters and our system, indicating that our developed system could assess tremor severity more objectively. According to our results, the S1 and S3 are suitable to be used as the tremor evaluation, because of the high reliability, high correlation and low bias, especially by using the *|*dr/dθ*|* in S1 test. In regular clinical practice, each patient’s spiral drawing would only be evaluated by one clinician (rater), potentially with bias. We conducted our study using the VRS values rated by three independent clinicians to eliminate bias, and increase reliability and objectiveness of the results. The variance of VRS could also be affected by the number of the raters. Therefore, future studies with additional data either from more patients or raters could improve the outcomes of our system.

There are some recent studies focusing using developing quantitative methods and employing tablets to gather detailed information from spiral drawings [5, 12, 13]. In addition to having used fast Fourier Transform (FFT) to analyze drawing characteristics in studies in the past decade, static and dynamic unraveling and empirical mode decomposition based methods were also proposed [5]. Moreover, using digital platform was found to provide more precise and robust results than clinical visual ratings [12]. To take advantage of previous findings, we further introduced different drawing paradigms and quantitative methods, and compared the results in different paradigms and linked them to the clinical evaluations, which was rarely discussed in the literature. In this study, we demonstrated the feasibility of using digital graphic tablets with different guiding paradigms to quantify the temporal and spatial characteristics of tremors, and the assessments were performed automatically. We may need more data from patients in a wider severity spectrum to obtain more accurate correlations with clinical assessments or to introduce new paradigms. A previous study suggests that the velocity of spiral drawing should be controlled by the patient with a constant angular speed as closed as possible to one turn per 2 sec [18]. However, no direct evidence is available on how and what the speed should be set to obtain the optimal clinical assessments. In our study, the guiding speed in test S3 was constant at 30 mm/s. Although we did not test any other guiding speeds in S3, in other words, we could not quantify the effect of the different speed, the programmed guiding point could control the drawing speed more precisely than current clinical practice [18]. In addition, our paradigm can also examine potential deficits in the eye–hand coordination using spiral drawings [19]. Our clinical assessments of the testing group were based on the scores rated independently by the three experienced neurologists. Although the subjectiveness during assessments of the clinical staff cannot be excluded, fairly high correlation coefficients were obtained, indicating the clinical relevance of the developed parameters. Moreover, we attempted to introduce simpler spiral drawing indices for easier and real-time tremor severity evaluation in clinical environments. Furthermore, interrater clinical scale comparison was performed thereafter.

## Conclusions

Using a digital graphic tablet, we developed a tremor assessing system with three different guiding paradigms for spiral drawing. The test “traced along a given spiral” is recommended due to its good interrater reliability. Three parameters were calculated to quantify temporal and spatial characteristics of the tremor and determine tremor severity from the spiral drawing. The reliability of our developed system was established by the clinical evaluations from three independent experienced neurologists.

## Additional file

Additional file 1: Datasets. (XLSX 286 kb)

## Abbreviations

AUC: Area under curve
ET: Essential tremor
PD: Parkinson’s disease
VRS: Visual rating scale
